# HE4 Might Participate in Extracellular Matrix Remodeling in Ovarian Cancer via Activation of Fibroblasts

**DOI:** 10.32604/or.2025.069007

**Published:** 2025-12-30

**Authors:** Yimin Liu, Bin Liu, Huabin Gao, Jinlong Wang, Jingya Duan, Xiaolan Huang, Yuexi Liu, Ying Huang, Wenjing Liao, Ruonan Li, Hua Linghu

**Affiliations:** 1 Department of Gynecology, The First Affiliated Hospital of Chongqing Medical University, Chongqing, 400016, China; 2 Department of Pathology, The First Affiliated Hospital of Chongqing Medical University, Chongqing, 400016, China; 3 Department of Pathology, College of Basic Medicine, Chongqing Medical University, Chongqing, 400016, China; 4 Molecular Medicine Diagnostic and Testing Center, Chongqing Medical University, Chongqing, 400016, China; 5 Department of Gynecology, The Second Affiliated Hospital of Chongqing Medical University, Chongqing, 400010, China

**Keywords:** Ovarian cancer, extensive extracellular matrix (ECM), fibroblast, human epididymis protein 4 (HE4), α-smooth muscle actin (α-SMA)

## Abstract

**Objectives:**

High-grade serous ovarian cancer (HGSOC), the most common subtype of epithelial ovarian cancer (EOC), exhibits a mesenchymal phenotype characterized by fibrotic stroma and poor prognosis. Human epididymis protein 4 (HE4), a key diagnostic biomarker for ovarian cancer, is involved in fibrotic processes in several non-malignant diseases. Given the clinical significance of stromal fibrosis in HGSOC and the potential link between HE4 and fibrosis, this study aimed to investigate the role of HE4 in the formation of stromal fibrosis in HGSOC.

**Methods:**

A total of 126 patients with gynecological conditions were included and divided into normal, benign, and EOC groups. Tissue stiffness was quantitatively measured and analyzed for its correlation with clinicopathological features. We further investigated the correlation between tumor stiffness and the expression levels of HE4 and fibroblast activation markers (α-smooth muscle actin (α-SMA) and fibroblast activation protein (FAP)) in tumor tissues from 22 HGSOC patients. *In vitro*, primary fibroblasts were treated with recombinant HE4 (rHE4) or conditioned media from HE4-knockdown ovarian cancer cells to assess fibroblasts activation and matrix contractility (Collagen gel contraction assays). *In vivo*, a subcutaneous xenograft model using HE4-knockdown cells was established to evaluate the effects of HE4 suppression on tumor growth and extensive extracellular matrix (ECM) remodeling.

**Results:**

Ovarian cancer tissues showed significantly increased stiffness compared to benign/normal groups, showing positive correlation with serum HE4 levels. High-stiffness HGSOC tumors exhibited upregulated expression of HE4, α-SMA, FAP, and collagen I. rHE4 stimulated fibroblast activation and enhanced matrix contractility, whereas HE4 knockdown in cancer cells abrogated these pro-fibrotic effects. *In vivo*, HE4-silenced xenografts displayed restricted tumor growth accompanied by reduced stromal expression of α-SMA, FAP, and collagen I.

**Conclusion:**

Our findings suggest that HE4 may facilitate ECM remodeling in HGSOC through promoting fibroblast activation and increasing collagen deposition.

## 1 Introduction

Ovarian cancer remains one of the most lethal gynecologic malignancies, with HGSOC being the most prevalent histopathological subtype [1,2]. More than 75% of HGSOC patients are diagnosed at an advanced stage, often presenting with extensive peritoneal metastases and substantial ascites [3]. According to gene expression profiles and tumor microenvironmental features identified by The Cancer Genome Atlas (TCGA), HGSOC can be classified into four molecular subtypes: mesenchymal, immunoreactive, differentiated, and proliferative [4,5]. Among these, the mesenchymal subtype is linked to poorer clinical outcomes, including shorter overall survival and increased resistance to platinum-based therapy [6,7]. A hallmark of the mesenchymal subtype of HGSOC is pronounced desmoplasia, characterized by abundant infiltration of α-SMA-positive fibroblasts and excessive ECM deposition [4,8]. This highly fibrotic tumor microenvironment is frequently associated with increased tissue stiffness, which significantly influences tumor biology [8,9]. Mechanistically, tissue stiffness promotes 3D collective invasion in breast cancer via the YAP-POSTN-integrin pathway [10]. Similarly, in oral squamous cell carcinoma (OSCC), stiffness accelerates progression through cancer-associated fibroblasts (CAFs)-derived LOX/FAK signaling [11]. Critically, in ovarian cancer, ECM stiffening induces platinum resistance via integrin-FAK-YAP activation [12]. These findings collectively underscore mechanical cues as pivotal drivers of progression and therapy resistance. Therefore, elucidating the origins of tissue stiffening in mesenchymal-type HGSOC and identifying fibrosis-specific therapeutic targets could provide novel strategies to disrupt pro-tumorigenic mechanosignaling and improve outcomes.

HE4, encoded by the *WFDC2* gene, is a well-established biomarker for ovarian cancer diagnosis and monitoring, with elevated serum levels strongly correlating with poor clinical outcomes [13,14]. Beyond its diagnostic utility, HE4 promotes ovarian tumorigenesis, chemoresistance, and metastasis [15,16]. Emerging evidence implicates HE4 in fibrotic pathogenesis: In renal fibrosis, it drives pathological ECM accumulation via protease inhibition, specifically by blocking MMP-2 activity [17]. Clinically, serum HE4 elevation parallels disease severity in cystic fibrosis (CF) and idiopathic pulmonary fibrosis (IPF), with concurrent upregulation in fibrotic lesions [18,19]. Although HE4 appears to play a critical role in fibrotic processes in multiple non-malignant diseases, HE4’s functional impact on cancer-associated fibrosis remains uncharacterized.

This study aims to elucidate the mechanistic role of human epididymis protein 4 (HE4) in ECM remodeling during ovarian cancer progression. We analyzed clinical associations between serum HE4 levels, tumor stiffness, and stromal fibroblast activation markers; evaluated the direct effects of recombinant HE4 on fibroblast activation and matrix contractility *in vitro*; explored the regulatory role of tumor cell-derived HE4 on fibroblast activation; and assessed the impact of HE4 suppression on ECM remodeling and tumor growth in subcutaneous xenograft models.

## 2 Methods and Materials

### 2.1 Clinical Study

This retrospective cohort study was conducted at the First Affiliated Hospital of Chongqing Medical University. The specimens were derived from 143 gynecological patients who underwent oophorectomy at the First Affiliated Hospital of Chongqing Medical University between August 2023 and January 2025. Clinical information and baseline serum HE4 levels were collected from the medical record system. The inclusion criteria were as follows: (1) Diagnosed with gynecological diseases requiring surgical intervention; (2) preoperative blood sampling for serum HE4 levels; (3) signing an informed consent form for postoperative tissue stiffness measurements, pathological examination and consent to use the relevant information in this study. Exclusion criteria were applied as follows (n = 17): (1) ovarian germ cell tumors, sex cord-stromal tumor, carcinosarcomas and metastatic tumors of the ovary; (2) patients with renal dysfunction causing elevated levels of HE4; (3) specimens that did not meet the requirements for hardness measurements (radius **r** < 30 mm, thickness **h** < 12 mm). All patients provided their written informed consent. A total of 126 patients were included and stratified by histopathological diagnosis into three groups: normal ovarian controls (n = 26; excised during surgery for uterine fibroids or cervical carcinoma), benign ovarian disease (n = 30), and EOC cases (n = 70). The EOC group included 46 cases of HGSOC and 24 cases of other types of EOC. Based on the average tissue stiffness values, the HGSOC cohort was divided into low- (n = 24) and high-stiffness (n = 22) groups.

Fagotti scores were assessed during diagnostic laparoscopy to evaluate intra-abdominal tumor dissemination. The scoring system evaluates seven parameters, including omental cake, diaphragmatic carcinomatosis, mesenteric retraction, bowel and stomach infiltration, liver surface metastases, and peritoneal carcinomatosis, with a total score ranging from 0 to 14, as described by Fagotti et al. [20].

### 2.2 Hardness Measurement

The mechanical properties of *ex vivo* specimens were quantified using a digital Shore durometer (Model LX-C; San liang Measuring Tools Co., Ltd., Dongguan, China). This hardness tester employs a 0–100 HC measurement range with 0.5 HC resolution, specifically calibrated for evaluating viscoelastic materials including biological soft tissues. Specimens were subjected to hardness testing within 30 min post-excision in operation room. Using a 2.5 mm diameter spherical indenter, five equidistant measurement points were selected on the intact external surface. Hardness counts were recorded at five different locations and the mean value was calculated as the representative hardness parameter for each specimen.

### 2.3 Fibroblast Isolation and Characterization

Normal omental fibroblasts (NOFs) were isolated from female patients (n = 10) undergoing elective laparoscopic surgery for non-cancerous gynecological conditions. After removal of blood vessels and adipose tissue with ophthalmic scissors and forceps, tissue fragments (1 mm^3^) were placed on 10 cm cell culture dishes and incubated at 37°C in a humidified atmosphere (5%, CO_2_). Characterization of NOFs: NOFs were distinguished by high expression of vimentin and lack of the epithelial marker cytokeratin 7 (CK7).

### 2.4 Ethical Statement

All procedures were conducted in accordance with the World Medical Association Declaration of Helsinki (2023 edition) for ethical conduct in human research. The research protocol was approved by the Ethics Committee of the First Affiliated Hospital of Chongqing Medical University (Approval No.: k2023-435).

### 2.5 Cell Culture

Human high-grade serous adenocarcinoma OVCAR3 and OVCAR8 cells were cultured in Roswell Park Memorial Institute (RPMI) 1640 (Sigma, R6504, Saint Louis, MI, USA) medium containing 10% fetal bovine serum (FBS) (Pricella, 164210-50, Wuhan, China) and 1% penicillin and streptomycin (Pricella, C0222). 293 T cells were cultured in Dulbecco’s Modified Eagle’s Medium (DMEM) (Pricella, PM150210) containing 10% FBS and 1% penicillin and streptomycin. NOFs were cultured in Dulbecco’s Modified Eagle Medium/Nutrient Mixture F-12 (DMEM-F12) (Pricella, PM150312) containing 12% FBS and 1% penicillin and streptomycin. In some experiments, NOFs were cultured with rHE4 (MCE, HY-P71432, Monmouth Junction, NJ, USA)-DMEM-F12 mixture (3 μg/mL) or conditioned medium from HE4-knockdown OVCAR3/OVCAR8 cells. Induced CAFs were generated by coculture of NOFs with ovarian cancer cells. Cells were cultured in a 37°C and 5% CO_2_ incubator. All cell lines were authenticated by tandem repeat sequence analysis and routinely tested Mycoplasma-negative.

### 2.6 Plasmids and Expression

The shRNA target sequences, sh-HE4-1: CCGGGGCTGACCAGAACTGCACGCAACTCGAGTTGC- GTGCAGTTCTGGGTCAGCTTTTTT, sh-HE4-2: CCGGTTCGGGCTTCACCCTAGTCTCACTCG- AGTGAGACTAGGTGTGAAGCCGAGAAGTTTTT and sh-HE4-Negative Control (NC): CCGG-GGTTCTCCGAACGTGTCACGT-CTCGAG-ACGTGACACGTTCGGAGAACC-TTTTTG, which were designed by Tsingke Biotechnology Co., Ltd. (Xi’an, China), and made to clone them into the PLKO.1 backbone. 293 T cells were inoculated at 1 × 105 per well in 6-well plates in DMEM medium containing 10% FBS for lentiviral preparation. After 12 h, 2 μg of expression vector, 1.25 μg of pSPAX2, and 0.75 μg of pLP/VSV-G packaging vector were used to transfect the cells. Viral supernatants were collected at 48 and 72 h post-transfection, filtered through a 0.22 μm filter (Nest, 331011, Wuxi, China) and added to OVCAR3 and OVCAR8 cell culture dishes for 72 h, and then screened with puromycin (5 μg/mL). As a control, lentivirus containing a random sequence shRNA was used. The transfection efficiency was verified by western blot analysis, and the shRNA knockdown cells obtained were screened for subsequent experiments.

### 2.7 Western Blotting

Total Protein Extraction: Total protein was extracted from tumor tissues and cultured cells (OVCAR3/8 and primary fibroblasts). Approximately 30 mg of frozen tumor tissue was homogenized in RIPA lysis buffer (Beyotime, P0013B, Shanghai, China) containing 1% PMSF (Beyotime, ST506) at a 1:10 (w/v) tissue-to-buffer ratio, with three 2 mm zirconia beads, using a bead mill (JINGXIN, JXFSTPRP-CL, Shanghai, China) at 80 Hz for 10 cycles (5 s on/5 s off, 4°C). Cells at 80%–90% confluence were washed with 4–5 mL PBS (0.01 M, pH: 7.4) and lysed in the same buffer. All lysates were incubated on ice for 15 min, ultrasonicated (SCIENTZ, Scientz-IID, Zhejiang, China; 80 W, 12 cycles, 3 s on/3 s off), and incubated for another 15 min on ice. Samples were then centrifuged at 12,000× *g* for 15 min at 4°C (Thermo Fisher, ST 16R, Bremen, Germany). Supernatants were collected for Bicinchoninic Acid Assay (BCA) protein quantification. The remaining supernatants were mixed with 5× loading buffer (Beyotime, P0010) at 1:4 ratio, denatured at 100°C for 5 min, and stored at −20°C.

Protein Quantification: Protein concentrations were determined using the BCA assay kit (Beyotime, P0010). Samples were incubated with working reagent (37°C, 30 min) and measured at 562 nm (Tecan, Infinite M PLEX, Männedorf, Swiss).

Total protein lysates underwent separation via sodium dodecyl sulfate polyacrylamide gel electrophoresis. In brief, protein samples were electrophoresed at 80–120 V under a constant electrical current for 60–120 min. Subsequently, the proteins were transferred to a polyvinylidene fluoride membrane (Millipore, Burlington, MA, USA) and blocked with 5% skimmed milk at 37°C for 2 h. The membrane was then incubated overnight at 4°C with primary antibodies HE4 (1:1000; Proteintech, 66557-1-Ig, Wuhan, China), α-SMA (1:1000; Proteintech, 14395-1-Ig), FAP (1:800; HUABIO, ET1704-23, Hangzhou, China), Collagen I (1:800; Proteintech, 67288-1-Ig), Vimentin (1:2000; HUABIO, ET1610-39) and GAPDH (1:10,000; Proteintech, 60004-1-Ig). Membranes were washed with TBST(20 mM Tris, 137 mM NaCl, 0.1% Tween-20, pH 7.4) buffer, prepared by adding 0.1% (v/v) Tween-20 (BioFroxx, 1247ML100, Hesse, Germany) to 1× TBS solution reconstituted from powder (Servicebio, G0001-15, Wuhan, China) and incubated with HRP-conjugated secondary antibodies (1:10,000; Boster, Wuhan, China) at 37°C for 1 h. Signals were detected using ECL luminescence (KeyGEN BioTECH, KGC4602-100, Nanjing, China) and exposed in a Bio-Rad imaging system (BIO-RAD, V3 Western Workflow, Hercules, CA, USA).

### 2.8 Cellular Immunofluorescence

Cells (primary fibroblasts) adhered to slides were fixed with 4% paraformaldehyde (Biosharp, BL539A, Hefei, China), permeabilised with an immunostaining potent permeabilising solution (Beyotime, P0097). After blocking with goat serum albumin (Boster, AR0009) for 30 min at room temperature, then 1× PBS (pH: 7.4) diluted anti-α-SMA (1:500; Proteintech, 14395-1-AP), anti-cytokeratin7 (1:400; HUABIO, R1309-4), anti-Vimentin (1:800; HUABIO, EM0401) primary antibody mixture was added, and the cells were incubated overnight at 4°C. Cells were washed three times with PBS. Cells were incubated with DyLight 594 (1:400; Abbkine, A23420, Wuhan, China) and DyLight 488 (1:400; Abbkine, A23210) for 1 h at room temperature, and the nuclei were stained with DAPI (Boster, AR1176) for 5 min and washed three times with PBS. The slices were sealed with a droplet of anti-fluorescence quenching sealer (Beyotime, P0126), and images were acquired using a confocal microscope (Zeiss, LSM 800, Oberkochen, Germany).

### 2.9 Tissue Immunohistochemistry

5 μm Formalin Fixed Paraffin Embedded (FFPE) sections were deparaffinized in xylene (undiluted, 100%) and rehydrated through graded ethanol. Antigen retrieval was performed using EDTA antigen repair buffer (pH 9.0, Servicebio, G1203) at 95°C for 20 min. Sections were treated with 3% H_2_O_2_ for 20 min, blocked with goat serum for 30 min, and then incubated with anti-HE4 (1:800; Proteintech, 66557-1-Ig), anti-α-SMA (1:800; Proteintech, 14395-1-AP), anti-FAP (1:200; HUABIO, ET1704-23), anti-Collagen I (1:1000; Proteintech, 67288-1-Ig) antibodies at 4°C overnight. The following day, the slices were incubated with ready-to-use horseradish peroxidase (HRP)-conjugated goat anti-rabbit/mouse secondary antibodies (undiluted, as per manufacturer’s protocol; Zhongshan Goldenbridge, PV9000, Beijing, China) for 30 min at room temperature, and stained using DAB (Zhongshan Goldenbridge, ZLI-9017). Nuclei were stained blue using haematoxylin (ready-to-use, Zhongshan Goldenbridge, ZLI-9610). The sections were then dehydrated, cleared with xylene (undiluted, 100%), and mounted. Images were acquired using a digital histopathology system (Zeiss, Axio Imager A2, Oberkochen, Germany).

### 2.10 Collagen Contractility Assay

96-well plates were coated with 2% BSA solution prepared from BSA powder (Solarbio, A8010, Beijing, China) dissolved in PBS (0.01 M, pH: 7.4, Servicebio, G4202-500 mL) and filter-sterilized through a 0.22 μm filter (Nest, 331011). Cells (primary fibroblasts) were dissociated with 0.25% trypsin (Servicebio, G4001-100 mL), diluted to 1 × 10^6^ cells/mL in growth medium. 100 μL of the diluted cells were combined with 100 μL of rat tail collagen (Corning, 354236, Corning, NY, USA) working solution. This mixture was then transferred to the prepared 96-well plates. Following a 30-min incubation period, the collagen gel was detached from the well edges using a pipette tip, and 200 μL of cell culture medium was added. Gel contraction was assessed via photography at 48 h, and collagen area was quantified using ImageJ software (National Institutes of Health; version 1.53t, Bethesda, MD, USA).

### 2.11 Xenograft Model of Ovarian Cancer

Ten 5-week-old female Balb/c nude mice (Vital River, Beijing, China) were randomly divided into two groups (n = 5 per group) and subcutaneously injected with either OVCAR3-HE4-MOCK or OVCAR3-HE4-knockdown cells (5 × 10^6^ cells/100 μL PBS (0.01 M, pH: 7.4)). Randomization was performed using a computer-generated random number sequence to minimize allocation bias. The sample size was calculated using G*Power software (version 3.1; Heinrich-Heine-Universität Düsseldorf, Düsseldorf, Germany) based on a medium effect size (Cohen’s d = 0.8), 80% power, and a significance level of 0.05, resulting in a minimum of 5 mice per group. Tumor growth was monitored every two days by caliper measurements, and body weight was recorded concurrently. Tumor volume was calculated as $V=ab^{2}/2$, where *a* is the maximum and *b* the minimum tumor diameter. Tumors became palpable by day 7 post-injection. Formalin-fixed tumors were paraffin-embedded, sectioned at 5 μm, and subjected to immunohistochemical staining. All animal procedures were approved by the Animal Care and Use Committee of Chongqing Medical University (Approval No.: IACUC-CQMU-2025-0212).

### 2.12 Statistical Analysis

All statistical analyses and data visualizations, including violin plots, were performed using GraphPad Prism 8.0 (GraphPad Software, San Diego, CA, USA). Categorical variables were expressed as frequencies and percentages (%), and differences between groups were assessed using Pearson’s chi-square test or Fisher’s exact test depending on the expected frequencies (Pearson’s test when all expected frequencies ≥5; Fisher’s exact test when any expected frequency <5). Data normality was evaluated using the Shapiro-Wilk test (α = 0.05), and homogeneity of variances was assessed by Levene’s test. Normally distributed data with equal variances were presented as mean ± standard deviation (SD), and comparisons between two groups were performed using Student’s *t*-test; for three or more groups, one-way analysis of variance (ANOVA) followed by appropriate post hoc multiple comparison tests (e.g., Bonferroni or Tukey’s test) was used. For non-normally distributed data, values were expressed as median and interquartile range (IQR), and group differences were analyzed using the Mann-Whitney U-test (two groups) or Kruskal-Wallis H-test (more than two groups). Correlations between ordered variables were assessed using Spearman’s rank correlation coefficient. All statistical tests were two-sided, and *p*-values < 0.05 were considered statistically significant unless adjusted for multiple comparisons. All experiments were performed independently at least three times.

## 3 Results

### 3.1 Association of Tissue Stiffness with Patient Clinical Characteristics

**Figure 1:**
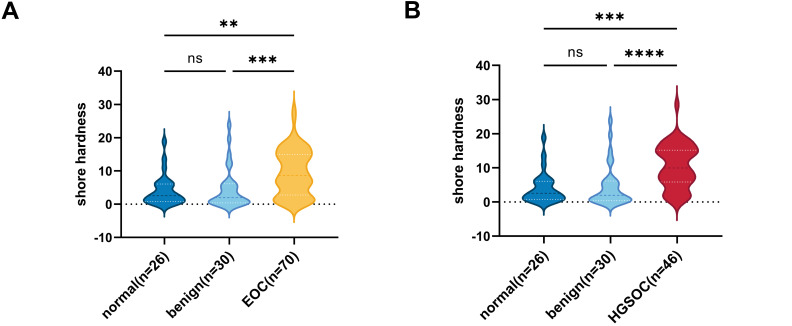

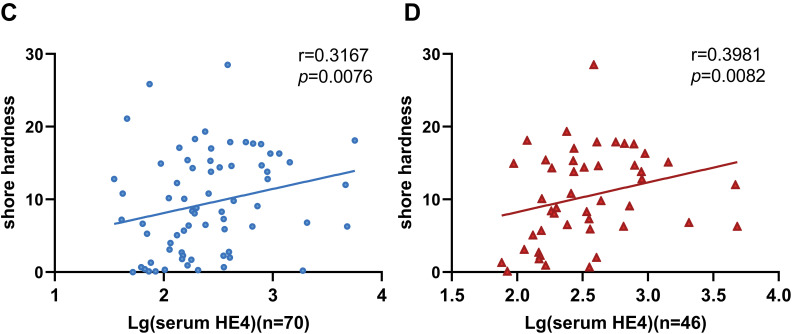
The correlation between ovarian tissue stiffness and clinical characteristics. **(A)** Shore hardness values for normal, benign, and malignant ovarian tissues. **(B)** Violin plots showing HGSOC tissues hardness compared to both benign ovarian disease and normal tissues. **(C)** The correlation between serum HE4 levels and ovarian tissue stiffness in EOC (n = 70). **(D)** The correlation between serum HE4 levels and tissue stiffness in HGSOC patients (n = 46). **p < 0.01, ***p < 0.001, ****p < 0.0001, ns = not significant

Previous studies have demonstrated that tumor tissues exhibit higher stiffness than normal tissues [21]. To investigate the relationship between tissue stiffness and ovarian pathology, we collected clinical data from 126 patients and classified them into three groups based on histopathological diagnosis: normal ovary (n = 26), benign ovarian disease (n = 30), and EOC (n = 70). Tissue stiffness was measured using a durometer. Baseline characteristics of the patients are summarized in Table 1, and detailed characteristics of EOC subtypes are provided in Table S1. Tissue stiffness was significantly higher in EOC tissues than in both normal and benign groups, with no significant difference between the normal and benign groups (Fig. 1A). Notably, the stiffness difference between HGSOC and the normal/benign groups was more pronounced than that observed in the overall EOC group (Fig. 1B).

**Table 1:** Clinicopathologic features of the patients

Characteristics	Normal	Benign	EOC	*p* Value
	(n **=** 26)	(n **=** 30)	(n **=** 70)	
**Age (years)**	**52.92 ± 8.49**	50.23 ± 16.29	56.11 ± 11.26	0.12
(mean ± SD)				
**BMI (kg/m** ^**2**^ **)**	23.74 (21.89, 25.29)	23.31 (21.50, 24.04)	24.04 (21.63, 25.65)	0.50
Median (IQR)				
**Menopausal status (n, %)**				0.18
Premenopause	15 (57.70%)	17 (56.70%)	51 (72.90%)	
Postmenopause	11 (42.30%)	13 (43.30%)	19 (27.10%)	
**Histology (n, %)**				
Normal	26, 100%			
Serous/mucinous cystadenoma		10/7, 34%/23%		
Serous/mucinous borderline tumor		7/6, 23%/20%		
HGSOC				46, 65.71%
Other EOC				24, 34.29%
**Serum CA125 (U/mL)**	20.80 (14.85, 26.80)	21.00 (15.40, 83.10)	454.35 (170.18, 1594.10)	**<0.0001**
Median (IQR)				
**Serum HE4 (pmol/L)**	41.00 (34.00, 53.00)	38.00 (30.00, 47.00)	202.50 (119.25, 414.25)	**<0.0001**
Median (IQR)				
**Shore hardness**	2.55 (0.86, 5.88)	1.95 (0.40, 5.95)	8.62 (2.88, 14.87)	**<0.0001**
Median (IQR)				

Note: BMI, body mass index; EOC, epithelial ovarian cancer; HGSOC, high-grade serous ovarian cancer; CA125, cancer antigen 125; HE4, human epididymis protein 4.

Some studies have reported an association between HE4 and fibrotic progression in various diseases [22–24]. Quantitative analysis confirmed that serum HE4 levels were positively correlated with tissue stiffness in EOC, with a notably stronger association observed in HGSOC (Fig. 1C,D). This finding is consistent with the characteristic desmoplastic microenvironment of HGSOC. However, the correlation remains in a preliminary exploratory phase, and the relatively low *r* values may be affected by various factors, including sample size and experimental variability. These findings suggest that HE4 may serve as a biomarker for increased tissue stiffness in ovarian cancer and may contribute to the development of the mesenchymal phenotype in HGSOC.

### 3.2 Tissue Stiffness Correlates with Fibroblast Activation and HE4 Elevation in HGSOC

To further validate the association between HE4 expression and tissue stiffness in HGSOC, a cohort of 46 patients pathologically diagnosed with HGSOC was analyzed. Patients were classified into high-stiffness (n = 24) and low-stiffness (n = 22) groups based on the average tissue stiffness. Baseline clinical characteristics are presented in Table 2. It was found that patients in the high-stiffness group exhibited significantly higher Fagotti scores.

**Table 2:** Correlation analysis of tissue stiffness and clinical features in HGSOC

Characteristics	Low Stiffness (n **=** 24)	High Stiffness (n **=** 22)	*p* Value
**Age (years)**	58.00 ± 9.95	56.64 ± 8.12	0.62
(Mean ± SD)			
**BMI (kg/m** ^**2**^ **)**	23.41 ± 2.27	24.13 ± 4.00	0.47
(Mean ± SD)			
**Menopausal status (n, %)**			
Premenopause	8 (33.33%)	3 (13.64%)	
Postmenopause	16 (66.67%)	19 (86.36%)	
**FIGO stage (n, %)**			0.50
Stage II	5 (21.74%)	2 (9.09%)	
Stage III	13 (56.52%)	14 (63.64%)	
Stage IV	5 (21.74%)	6 (27.25%)	
**Fagotti Score**	6.00 (3.50, 8.00)	8.00 (8.00, 10.00)	**0.03**
Median (IQR)			
**Serum CA125 (U/mL)**	597.85 (305.00, 1465.50)	1542.8 (475.42, 3016.80)	0.07
Median (IQR)			
**Serum HE4 (pmol/L)**	195.50 (149.25, 371.75)	411.50 (268.75, 865.75)	**0.01**
Median (IQR)			

Note: HGSOC, high-grade serous ovarian cancer; BMI, body mass index; FIGO: International Federation of Gynecology and Obstetrics; CA125, cancer antigen 125; HE4, human epididymis protein 4.

**Figure 2:**
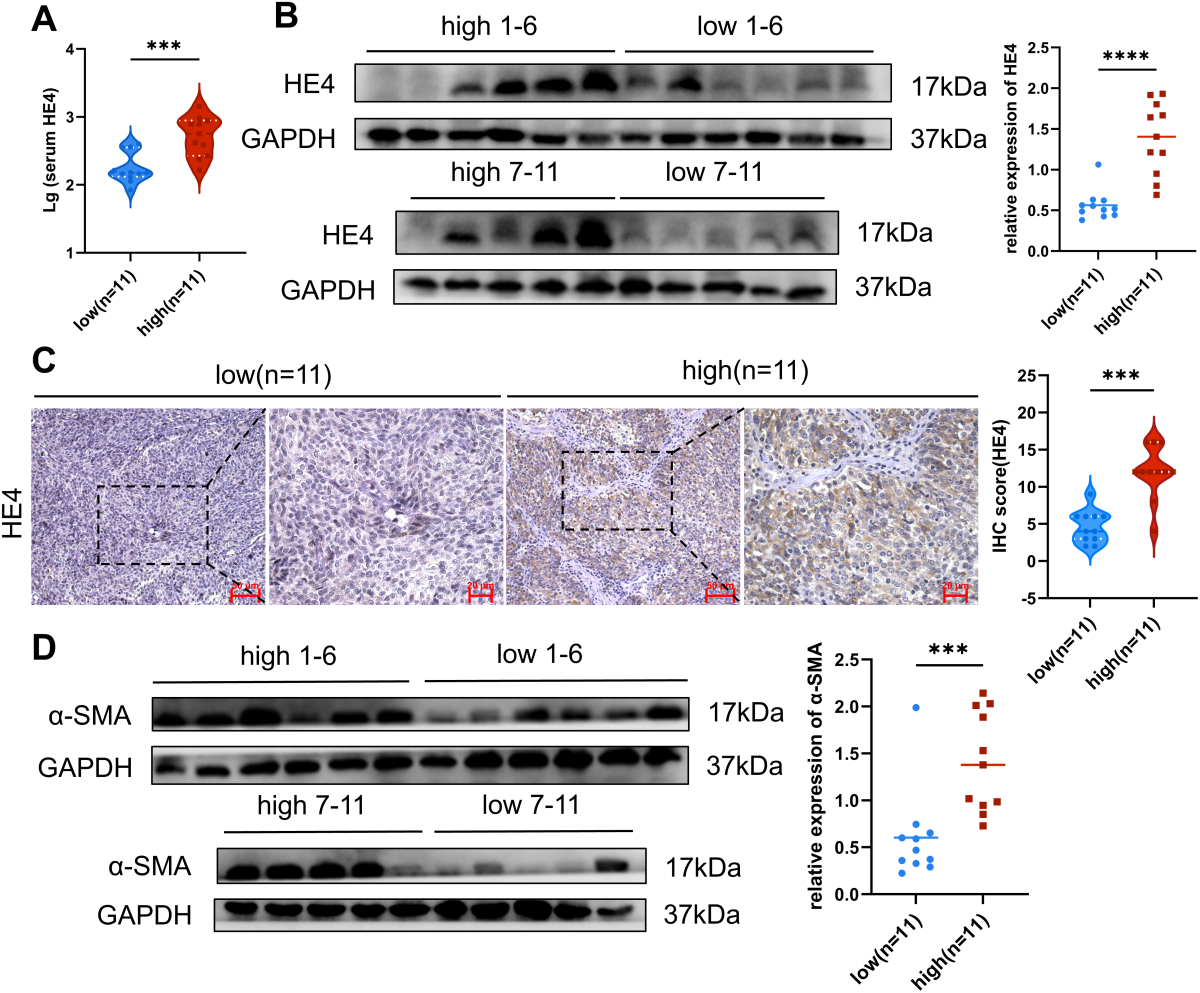

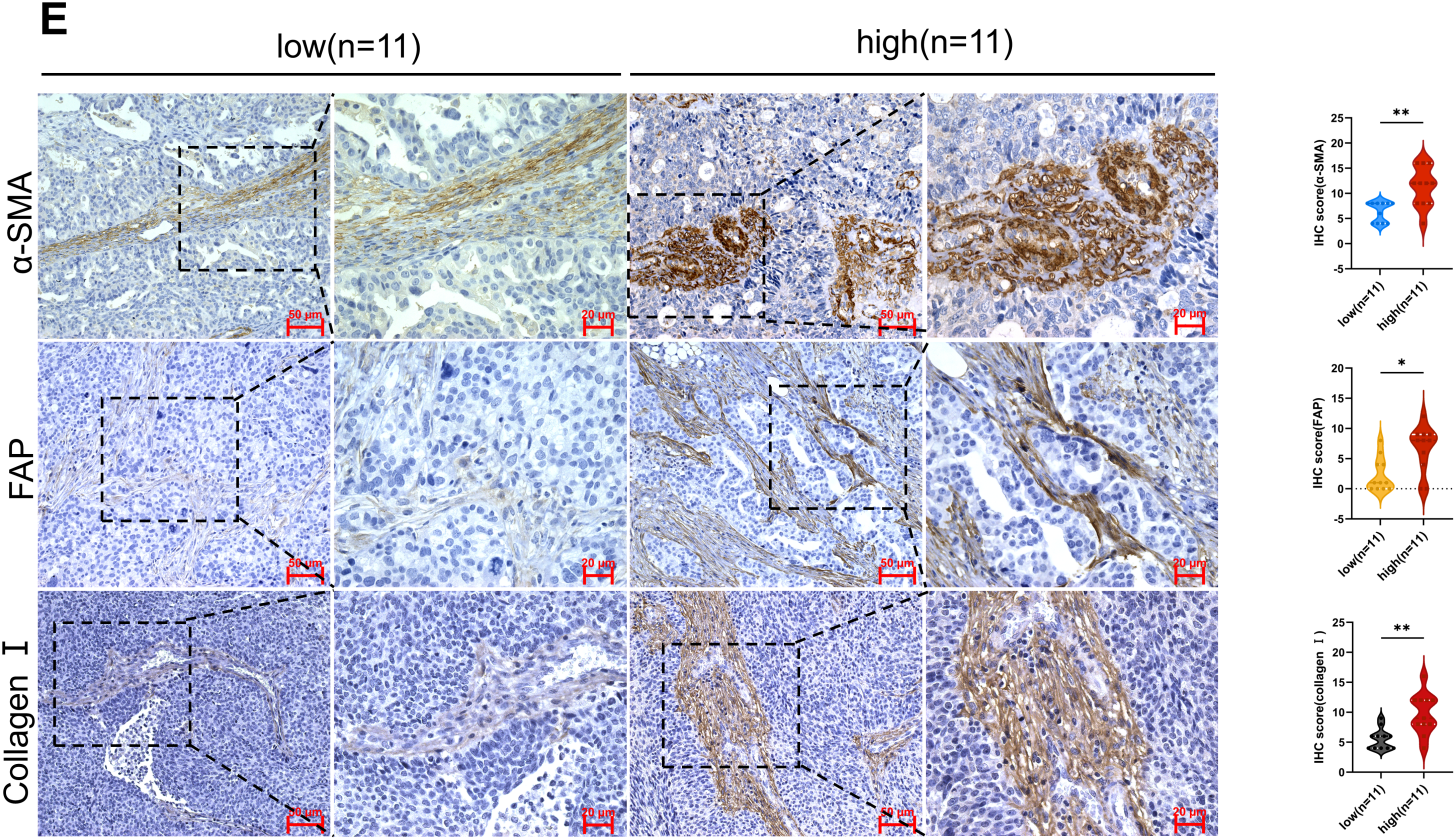
Expression of HE4 levels, fibroblast activation markers and collagen in high vs. low stiffness groups. **(A)** Serum HE4 levels in the high stiffness group and low stiffness group (n = 11/group). **(B)** The protein levels of HE4 in different stiffness groups were assayed by Western blot (n = 11/group). **(C)** Representative IHC staining of HE4 of cancer cell in high and low groups (n = 11/group), Scale bars, 50 μm/20 μm. **(D)** Western blot results show that the expression of fibroblast activation marker α-SMA (n = 11/group). **(E)** Immunohistochemistry results demonstrate fibroblast activation markers and Collagen I of mesenchymal areas in high and low groups (n = 11/group), Scale bars, 50 μm/20 μm. *p < 0.05, **p < 0.01, ***p < 0.001, ****p < 0.0001

To investigate the potential mechanisms underlying increased tissue stiffness, the expression levels of HE4, fibroblast activation markers, and type I collagen were assessed. Elevated serum HE4 levels were observed in the high-stiffness group (Fig. 2A), and increased HE4 expression in tumor tissues was confirmed by Western blot and immunohistochemistry, with stronger expression localized to tumor epithelial cells (Fig. 2B,C).

Activated CAFs, particularly myofibroblasts, have been recognized as the major ECM-producing cells in the tumor microenvironment [25]. In the high-stiffness group, elevated expression of α-SMA and FAP was detected (Fig. 2D,E), predominantly distributed in the tumor stroma. Moreover, a marked increase in type I collagen deposition was identified in high-stiffness tumor tissues (Fig. 2E). Collectively, these findings indicate that tissue stiffness may reflect the activation state of fibroblasts and the level of ECM production. The activation of CAFs and the ECM deposition are likely to be key driving factors of increased stiffness in ovarian cancer tissues.

### 3.3 Recombinant HE4 Promotes Fibroblast Activation

**Figure 3:**
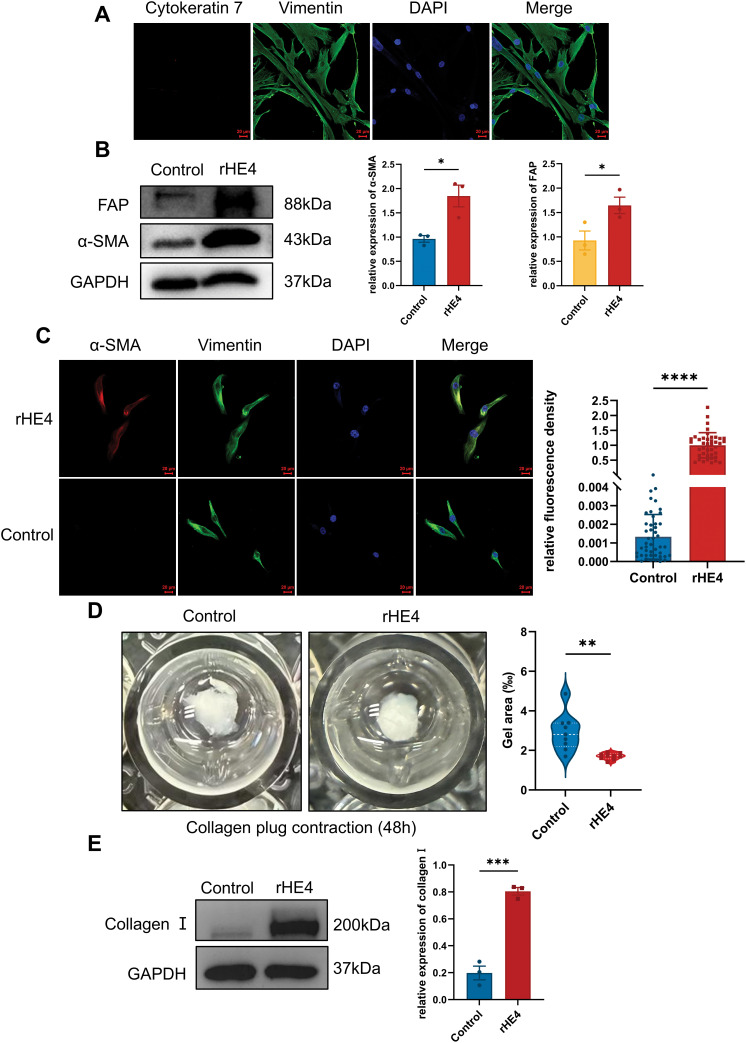
Effect of rHE4 treatment on fibroblast activation, fibroblast contraction and type I collagen synthesis. **(A)** Immunofluorescence shows the isolated NOFs with Vimentin staining and Cytokeratin 7 expression, with DAPI staining confirming the presence of cell nuclei. Scale bars, 20 μm. **(B)** Western blot analysis demonstrates the expression levels of fibroblast activation markers α-SMA and FAP in control and rHE4 treatment groups (3 μg/mL) (n = 3/group). **(C)** The expression of α-SMA and vimentin changes in NOFs cells under rHE4 treatment was further confirmed with immunofluorescence analysis (Control: n = 46, rHE4: n = 41). Scale bar, 20 μm. **(D)** Collagen contractility of NOFs control and treated with rHE4 (n = 9/group). **(E)** The ECM-related protein level of Collagen I by western blot (n = 3/group). *p < 0.05, **p < 0.01, ***p < 0.001, ****p < 0.0001

To determine whether HE4 directly promotes the transdifferentiation of normal fibroblasts into myofibroblasts, primary normal fibroblasts were isolated and characterized (Fig. 3A). Western blot analysis revealed that treatment with rhHE4 (3 μg/mL) significantly upregulated the expression of myofibroblast markers α-SMA and FAP compared to the control group treated with BSA (3 μg/mL) (Fig. 3B). This finding was further confirmed by immunofluorescence, which demonstrated that rhHE4 induced the transdifferentiation of normal fibroblasts into α-SMA–positive myofibroblasts (Fig. 3C).

Notably, the collagen gel contraction assay—a classical 3D model for evaluating fibroblast-mediated matrix remodeling—showed that fibroblasts treated with rhHE4 exhibited significantly enhanced contractile ability (Fig. 3D). In addition, expression of type I collagen, a key component of ECM, was markedly increased following rHE4 treatment (Fig. 3E).

### 3.4 HE4 Knockdown Inhibited Fibroblast Activation in Ovarian Cancer

**Figure 4:**
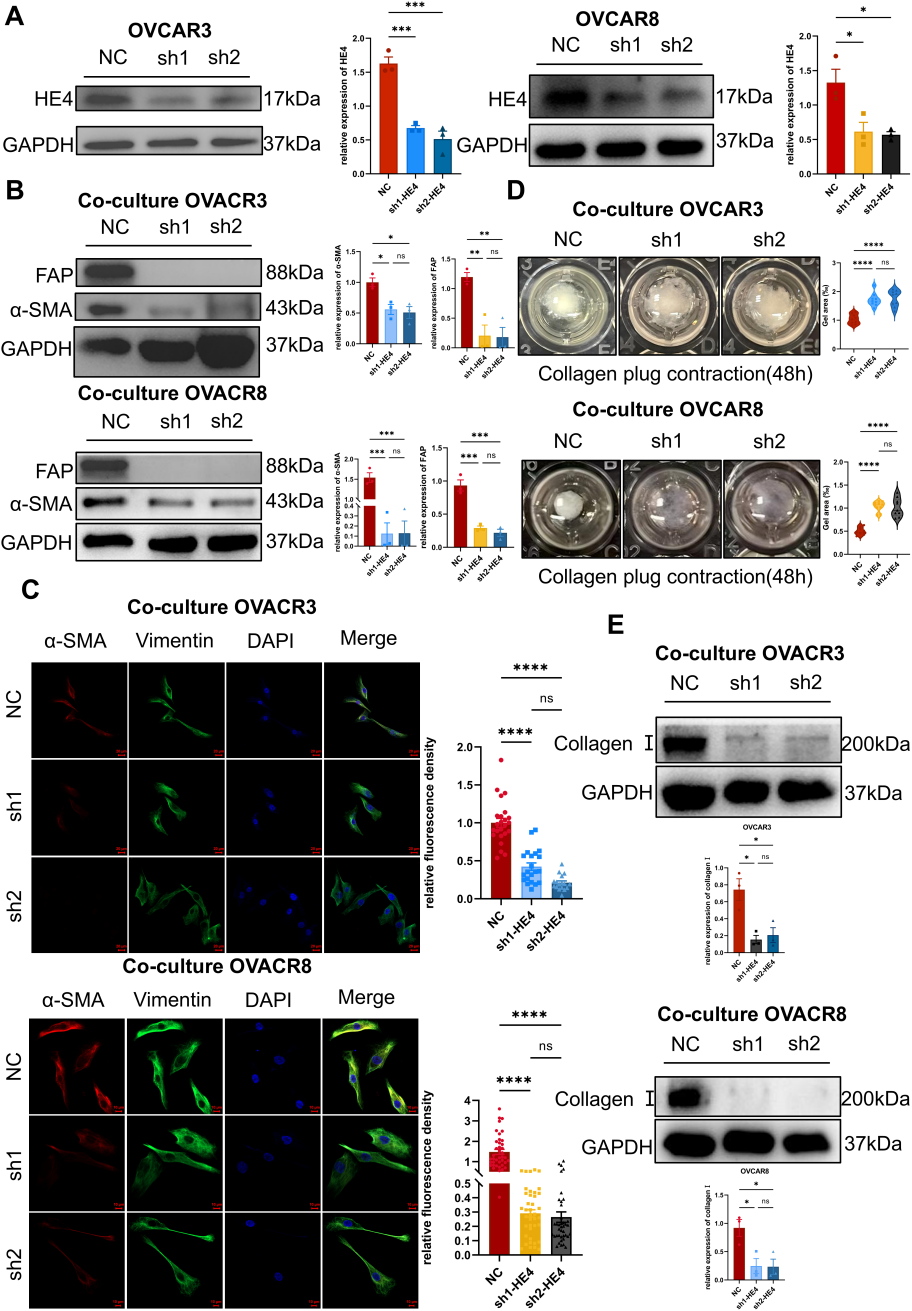
Cell-secreted HE4 induced fibroblast activation and extracellular matrix deposition. **(A)** HE4 knockdown cell lines were established in OVCAR3/OVCAR8 cells using lentiviral transfection, and the knockdown efficiency was verified by Western blot (n = 3/group). **(B)** Western blot analysis expression of α-SMA and FAP after NOFs were co-cultured with sh-HE4 or control groups of OVCAR3/OVCAR8 (n = 3/group). **(C)** The fibroblast markers of α-SMA and vimentin were analyzed by immunohistochemistry OVCAR3(NC: n = 24, sh1: n = 21, sh2: n = 17)/OVCAR8(NC: n = 40, sh1: n = 40, sh2: n = 40). Scale bar, 20 μm (OVCAR3)/10 μm (OVCAR8). **(D)** Effect of HE4 knockdown on collagen contractility OVCAR3/OVCAR8 (n = 9/group). **(E)** Treatment of NOFs co-cultured with the sh-HE4 groups led to decreased the level of Collagen I OVCAR3/OVCAR8 (n = 3/group). *p < 0.05, **p < 0.01, ***p < 0.001, ****p < 0.0001, ns = not significant

The effects of ovarian cancer cell–derived HE4 on fibroblast function were assessed through indirect co-culture, Western blotting, immunofluorescence, and collagen gel contraction assays. The WFDC2 gene, which encodes HE4, was silenced in the ovarian cancer cell lines OVCAR3 and OVCAR8 using shRNA (Fig. 4A). Conditioned media from the HE4-knockdown group (sh-HE4) and the negative control group (NC) were collected and indirectly co-cultured with normal fibroblasts.

Western blot analysis revealed that α-SMA and FAP expression levels were decreased in fibroblasts following co-culture with sh-HE4 cells (Fig. 4B). Immunofluorescence further demonstrated a reduction in α-SMA fluorescence intensity (Fig. 4C). Moreover, the collagen gel contraction capacity of fibroblasts was significantly reduced after treatment with sh-HE4 conditioned media (Fig. 4D). In addition, type I collagen expression in fibroblasts was inhibited by the sh-HE4 conditioned media (Fig. 4E).

These findings indicate that HE4 knockdown suppresses the ability of ovarian cancer cells to induce the transdifferentiation of normal fibroblasts into myofibroblastic cancer-associated fibroblasts (myCAFs).

### 3.5 HE4 Promotes Tumor Growth and Fibroblast Activation In Vivo

**Figure 5:**
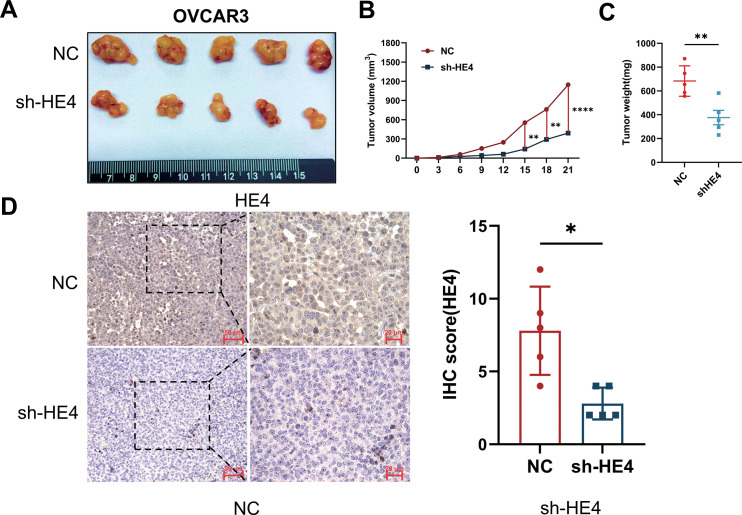

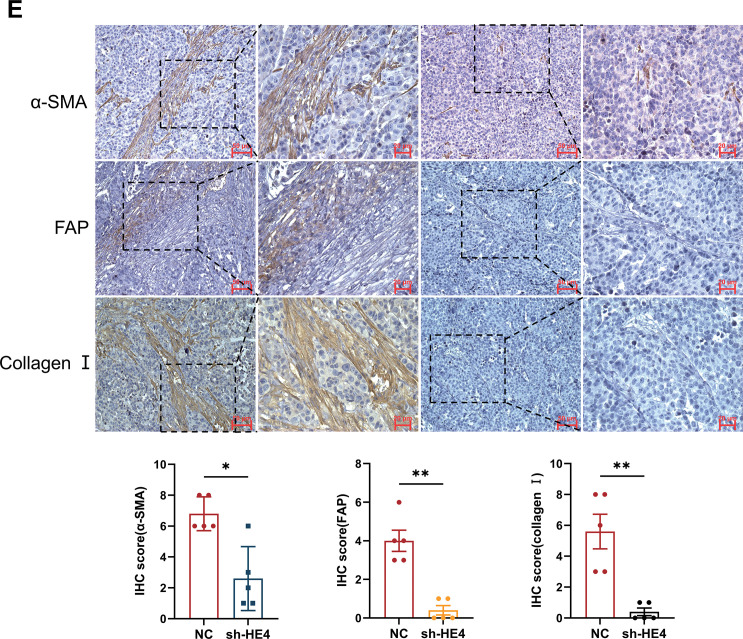
Fibroblast activation and collagen deposition were inhibited *in vivo*. **(A)** Representative images of tumors excised from mice injected with Normal Control (NC) or sh-HE4 OVCAR3 cells (n = 5/group). **(B)** Tumor volume was measured over 21 days, tumors in the HE4 knockdown groups grew significantly slower compared to the control group. **(C)** Tumor weight at the endpoint. Tumors in the HE4 knockdown groups were significantly lighter than those in the control group. **(D)** Representative IHC staining of HE4 and quantification of HE4-positive areas in NC and HE4 knockdown groups (n = 5/group). **(E)** Representative IHC staining of and quantification of fibroblast activation markers and Collagen I positive areas (n = 5/group). *p < 0.05, **p < 0.01, ****p < 0.0001

To further investigate the potential role of HE4 *in vivo*, subcutaneous xenograft tumor experiments were performed using OVCAR3 ovarian cancer cells with stable knockdown of WFDC2. Tumor volume and weight were found to be significantly reduced in the sh-HE4 group compared to controls (Fig. 5A–C), consistent with previous reports indicating that HE4 promotes ovarian cancer cell proliferation. Immunohistochemical analysis confirmed that HE4 expression was downregulated in the sh-HE4 group (Fig. 5D).

Meanwhile, the expression of fibroblast activation markers α-SMA and FAP was markedly decreased in the knockdown group, accompanied by a reduction in type I collagen deposition (Fig. 5E). These findings further support the critical role of HE4 in promoting the transdifferentiation of fibroblasts into cancer-associated fibroblasts in ovarian cancer.

## 4 Discussion

In this study, we revealed a novel functional role of HE4 in promoting stromal remodeling in HGSOC from a biomechanical perspective. Elevated HE4 levels were significantly associated with increased tumor stiffness and stromal fibrosis. Mechanistically, HE4 induces activation of fibroblasts into myCAFs phenotype and enhances type I collagen production, thereby promoting ECM remodeling and contributing to a stiffened tumor microenvironment.

HE4-mediated fibroblast activation likely alters the mechanical and biochemical properties of the stroma, which can facilitate tumor progression and therapeutic resistance. As major sources of ECM components, CAFs respond to signals such as TGF-β to drive matrix deposition and stiffening [26], which has been linked to enhanced proliferation, invasion, and drug resistance of tumor cells [27,28]. Our results demonstrate that HE4 inhibition significantly attenuates CAF activation and collagen accumulation, underscoring its potential as a therapeutic target for disrupting the fibrotic tumor stroma.

Interestingly, previous studies have demonstrated that HE4 plays a significant role in the fibrotic progression of various non-malignant diseases, including renal fibrosis, pulmonary fibrosis, and dilated cardiomyopathy [29–31]. For instance, Research indicates that HE4 activates the NF-κB pathway by promoting C3 and MMP2/9 expression, driving renal fibrosis [30]. Critically, under hypoxia, HE4 upregulation in renal cells activates the HIF-1α/HE4/NF-κB axis, inducing TIMP-1 to suppress MMP2 while initially promoting MMPs, collectively facilitating fibrotic deposition [32]. Similarly, in myocardial fibrosis, HE4 activates fibroblasts via ERK, inducing interstitial fibrosis [29]. These findings demonstrate conserved NF-κB/ERK mechanisms across tissues, further supported by 25-hydroxycholesterol’s NF-κB-dependent pro-fibrotic action resembling HE4 [33]. Together, these studies collectively suggest HE4’s critical role in fibroblast activation and ECM remodeling. By extending this profibrotic function to ovarian cancer, our findings provide new insights into conserved fibrotic mechanisms across pathological contexts. This positions HE4 as a potential therapeutic target for stromal reprogramming in ovarian cancer, with possible implications for other fibrosis-associated malignancies.

Preliminary evidence from this study indicates that HE4 inhibition suppresses fibroblast activation and collagen deposition, suggesting its involvement in regulating the fibrotic microenvironment of ovarian cancer. Given that HE4 has been reported as a fibrosis-associated biomarker in renal fibrosis, pulmonary fibrosis, and other fibrotic diseases [18,22,23], we hypothesize that HE4 may potentially serve as a biomarker for high-grade serous ovarian carcinoma (HGSOC) in the future, enabling dynamic quantification of tumor stromal fibrotic progression and therapy resistance risk. Based on the potential molecular mechanisms by which HE4 regulates stromal remodeling, further exploration of targeted intervention strategies may provide new perspectives for improving the tumor microenvironment and enhancing existing therapies.

Despite these promising findings, our study has certain limitations. Whether HE4 activates fibroblasts through canonical fibrotic pathways such as TGF-β/SMAD, RhoA/ROCK, or YAP/TAZ remains to be elucidated [34]. Moreover, the heterogeneity of CAF subsets poses a challenge in defining universal mechanisms. Future studies employing single-cell technologies and mechanistic dissection will be essential to unravel the complexity of HE4-mediated stromal remodeling and to refine its therapeutic targeting.

In summary, this study provides new evidence revealing a regulatory role of HE4 in stromal fibrosis of ovarian cancer from a biomechanical perspective. We demonstrate that HE4 promotes extracellular matrix remodeling by activating CAFs and enhancing type I collagen deposition, thereby potentially facilitating tumor progression. Collectively, our findings broaden the functional landscape of HE4 and highlight its potential as a therapeutic target for intervening in the ovarian cancer stromal microenvironment.

## Supplementary Materials



## Data Availability

The datasets generated or analyzed during the current study are available from the corresponding authors on reasonable request.

## Supplementary Materials

The supplementary material is available online at https://www.techscience.com/doi/10.32604/or.2025.069007/s1.

## Abbreviations

HGSOC: High-grade serous ovarian cancer
EOC: Epithelial ovarian cancer
HE4: Human epididymis protein 4
rHE4: Recombinant HE4
α-SMA: α-smooth muscle actin
FAP: Fibroblast activation protein
ECM: Extracellular matrix
TCGA: The Cancer Genome Atlas
OSCC: Oral squamous cell carcinoma
CAFs: Cancer-associated fibroblasts
CF: Cystic fibrosis
IPF: Idiopathic pulmonary fibrosis
NOFs: Normal omental fibroblasts
CK7: CYTOKERATIN 7
RPMI: Roswell Park Memorial Institute
FBS: Fetal bovine serum
DMEM: Dulbecco’s Modified Eagle’s Medium
DMEM-F12: Dulbecco’s Modified Eagle Medium/Nutrient Mixture F-12
myCAFs: Myofibroblastic cancer-associated fibroblasts
NC: Negative Control
BCA: Bicinchoninic Acid Assay
FFPE: Formalin Fixed Paraffin Embedded
ns: Not significant
HRP: Horseradish peroxidase
SD: Standard deviation
IQR: Interquartile range
BMI: Body mass index
CA125: Cancer antigen 125
FIGO: International Federation of Gynecology and Obstetrics
CCC: Clear cell cancer
LGSOC: Low-grade serous ovarian cancer
EC: Endometrioid cancer
MC: Mucinous cancer
