# Supplementary material for: HE4 Might Participate in Extracellular Matrix Remodeling in Ovarian Cancer via Activation of Fibroblasts
Source: Oncol Res. 2025 Dec 30;34(1):18. doi: 10.32604/or.2025.069007 (PMC12774540; doi:10.32604/or.2025.069007)
Supplement: Supplementary file 1 [file OncolRes-34-69007-s001.docx]

**Table S1. Characteristics of EOC Subtypes**

| Variable | HGSOC (n=43) | LGSOC (n=6) | CCC (n=6) | MC (n=5) | EC (n=7) |
| --- | --- | --- | --- | --- | --- |
| Age (years) |  |  |  |  |  |
| (Mean ± SD) | 57.5 ± 9.8 | 48.5 ± 17.5 | 56.5 ± 8.0 | 51.0 ± 17.7 | 59.3 ± 16.4 |
| BMI (kg/m²) |  |  |  |  |  |
| Median (IQR) | 23.2(21.1-24.9） | 25.0(22.9-27.4） | 25.4(24.3-26.5） | 24.5(21.3-25.0） | 25.2(21.4-25.9） |
| Serum CA125 (U/mL) |  |  |  |  |  |
| Median (IQR) | 606.0(336-1620） | 227.5(45-409） | 112.8(49-192） | 68.4(46-326） | 228.6(25-1910） |
| Serum HE4 (pmol/L) |  |  |  |  |  |
| Median (IQR) | 206(144-396） | 103(53-149） | 140(70-196） | 73(62-102） | 115(52-353） |
| Shore Hardness |  |  |  |  |  |
| Median (IQR) | 8.8(3.1-15.9） | 9.4(7.0-15.6） | 5.9(0.4-10.1） | 12.8(0.1-10.8） | 0.3(0.0-2.8） |
| FIGO Stage, n (%) |  |  |  |  |  |
| Stage I | 0 (0.0%) | 0 (0.0%) | 1 (16.7%) | 1 (20.0%) | 0 (0.0%) |
| Stage II | 6 (14.0%) | 2 (33.3%) | 2 (33.3%) | 1 (20.0%) | 4 (57.1%) |
| Stage III | 33 (76.7%) | 4 (66.7%) | 1 (16.7%) | 2 (40.0%) | 3 (42.9%) |
| Stage IV | 4 (9.3%) | 0 (0.0%) | 2 (33.3%) | 1 (20.0%) | 0 (0.0%) |
| Menopausal Status, n (%) |  |  |  |  |  |
| Premenopause | 25 (58.1%) | 2 (33.3%) | 4 (66.7%) | 4 (80.0%) | 4 (57.1%) |
| Postmenopause | 18 (41.9%) | 4 (66.7%) | 2 (33.3%) | 1 (20.0%) | 3 (42.9%) |

High Grade Serous Ovarian Cancer (HGSOC), Clear Cell Cancer (CCC), Low-Grade Serous Ovarian Cancer (LGSOC),

Endometrioid Cancer (EC), Mucinous Cancer (MC)
